# Combined Impact of Mean and Variability of Non-HDL Cholesterol on Myocardial Infarction in Hemodialysis Patients

**DOI:** 10.3390/jcm15010242

**Published:** 2025-12-28

**Authors:** Hanbi Lee, Ji Won Min, Tae Hyun Ban, Eun Sil Koh, Hye Eun Yoon, Young Soo Kim, Hyung Wook Kim, Byung Ha Chung

**Affiliations:** 1 Division of Nephrology, Department of Internal Medicine, Seoul St. Mary’s Hospital, College of Medicine, The Catholic University of Korea, Seoul 06591, Republic of Korea; hanbilee89@gmail.com (H.L.);; 2 Transplantation Research Center, College of Medicine, The Catholic University of Korea, Seoul 06591, Republic of Korea; 3 Department of Internal Medicine, Bucheon St. Mary’s Hospital, College of Medicine, The Catholic University of Korea, Seoul 14647, Republic of Korea; 4 Department of Internal Medicine, Eunpyeong St. Mary’s Hospital, College of Medicine, The Catholic University of Korea, Seoul 03312, Republic of Korea; 5 Division of Nephrology, Department of Internal Medicine, Yeouido St. Mary’s Hospital, College of Medicine, The Catholic University of Korea, Seoul 07345, Republic of Korea; 6 Department of Internal Medicine, Uijeongbu St. Mary’s Hospital, College of Medicine, The Catholic University of Korea, Seoul 11765, Republic of Korea; 7 Department of Internal Medicine, St. Vincent’s Hospital, College of Medicine, The Catholic University of Korea, Seoul 16247, Republic of Korea

**Keywords:** cardiovascular disease, cholesterol, renal dialysis, variability

## Abstract

**Objectives**: The aim of this study was to stratify cardiovascular risk based on the mean and variability of non-high-density lipoprotein cholesterol (non-HDL-C) in patients undergoing hemodialysis. **Methods**: Data were analyzed for 453 hemodialysis patients without a history of myocardial infarction (MI) or stroke, who underwent at least five lipid profile measurements at any one of seven university hospitals in the Republic of Korea between March 2009 and December 2020. Visit-to-visit non-HDL-C variability was calculated using the coefficient of variation. The endpoints of the study were newly diagnosed MI, stroke, or all-cause death. Patients were divided into four groups according to quartiles of the mean and variability of non-HDL-C. **Results**: During a median follow-up of 97.0 months, there were 39 cases of MI, 99 cases of stroke, and 96 deaths. The cumulative incidence rate of MI was significantly highest in the low mean/high variability group (log-rank *p* = 0.0296). However, there were no significant differences between groups in the incidence rates of stroke (log-rank *p* = 0.9939) or all-cause mortality (log-rank *p* = 0.9373). In the multivariable Cox regression analysis, age and low mean/high variability (HR: 3.311, 95% CI: 1.380–7.944) were identified as independent risk factors for MI. However, for stroke and all-cause mortality, age was the only independent risk factor. Moreover, neither the mean nor the variability of non-HDL-C alone was associated with MI, stroke, or all-cause mortality. **Conclusions**: Our results suggest that the coexistence of low mean non-HDL-C and high variability is associated with an elevated risk of MI in hemodialysis patients.

## 1. Introduction

Cardiovascular disease (CVD) constitutes the single largest contributor to death in end-stage kidney disease (ESKD) patients. In fact, mortality from CVD is 10–30-fold higher in ESKD patients than in the general population [1]. Altered lipid metabolism is a nontraditional risk factor for CVD in ESKD patients. However, despite the significance of lipid abnormalities, randomized controlled trials have not been successful in establishing a direct link between lowering low density lipoprotein cholesterol (LDL-C) through cholesterol-lowering treatment and reducing the incidence of major cardiovascular events [2,3,4,5]. The LDL-C level alone is not suitable for identifying ESKD patients who might benefit from cholesterol-lowering treatments.

Dyslipidemia and CVD in ESKD patients have characteristics and mechanisms distinct from those in the general population. In addition to quantitative abnormalities in lipid levels, dialysis patients frequently experience qualitative lipoprotein modifications driven by the uremic milieu or the dialysis process itself [6,7]. Furthermore, CVD in patients on dialysis can be influenced by non-atheromatous processes [8]. Therefore, cholesterol values alone might not be sufficient when evaluating cardiovascular risk in ESKD patients. The development of additional biomarkers to better discriminate CVD risk and identify patients who can benefit from available therapies is needed.

Serum non-high-density lipoprotein cholesterol (non-HDL-C) encompasses the cholesterol content of lipoprotein particles traditionally considered proatherogenic, such as LDL-C, lipoprotein(a), and triglyceride-rich lipoproteins [9]. Non-HDL-C is a predictor of cardiovascular risk and outcomes in the general population [10]. Additionally, a high visit-to-visit variability in cholesterol has been associated with fluctuations in atherosclerotic plaque composition and linked to cardiovascular events [11]. Therefore, variability in cholesterol levels might serve as a marker of residual cardiovascular risk among vulnerable patients.

The objective of this study was to identify biomarkers that can determine the level of cardiovascular risk in hemodialysis (HD) patients. Based on the above background, this study aimed to stratify cardiovascular risk considering both the mean and variability of non-HDL-C in patients undergoing HD.

## 2. Materials and Methods

### 2.1. Data Source and Study Population

All data were retrospectively extracted from the clinical data warehouse of the Catholic Medical Center (CMC), the largest healthcare network in Korea, which provided access to a consolidated electronic medical record database encompassing patients from the seven affiliated hospitals constituting the CMC, the Seoul, Bucheon, Eunpyeong, Incheon, Uijeongbu, Yeouido St. Mary’s, and St. Vincent’s Hospitals. We retrieved data for the following categories: demographics, comorbidities, medications, and laboratory findings.

**Figure 1:**
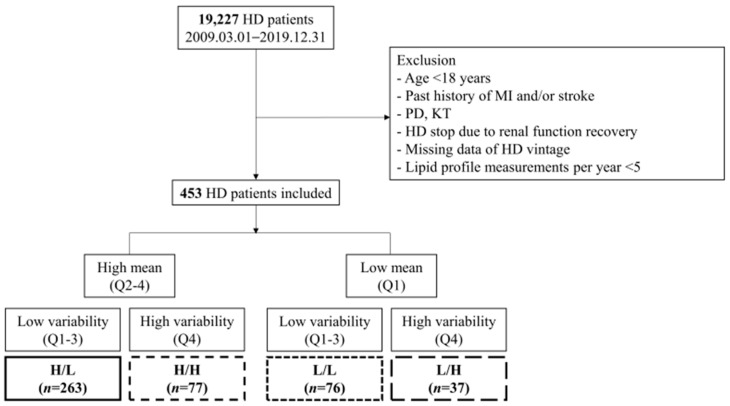
Flow chart of the study population. HD, hemodialysis; MI, myocardial infarction; PD, peritoneal dialysis; KT, kidney transplantation; Q, quartile; H, high; L, low.

Medical records of patients who underwent HD at CMC were retrieved for the period between 1 March 2009 and 31 December 2020. Of the initially identified 19,227 patients, we excluded those who did not undergo consecutive HD sessions for at least 90 days, were <18 years of age, had a past history of myocardial infarction (MI) [International Classification of Disease, 10th revision (ICD-10) codes: I21, I22] and/or stroke [ICD-10 codes: I63, I64], underwent peritoneal dialysis or kidney transplantation, stopped HD due to renal function recovery, had missing data for HD vintage, and had fewer than five lipid profile measurements per year. Ultimately, the study population consisted of 453 subjects (Figure 1). Due to institutional constraints, online hemodiafiltration was not performed at our center. Consequently, the analysis was restricted exclusively to patients undergoing HD.

### 2.2. Measurements and Definitions

Body mass index (BMI) was calculated as weight in kilograms divided by the square of height in meters. Diabetes mellitus (DM) was defined as the presence of at least one annual claim for ICD-10 codes E10–14, along with the prescription of antidiabetic medication. Similarly, hypertension was identified based on at least one annual claim for ICD-10 codes I10 or I11, accompanied by the prescription of antihypertensive agents. At our center, lipid profiles are assessed every 1 or 3 months, in accordance with medical insurance coverage. We calculated non-HDL-C by subtracting HDL-C from total cholesterol. Because non-HDL-C levels differ between males and females, we used sex-specific cutoff values. Visit-to-visit non-HDL cholesterol variability was defined using three indices: (i) coefficient of variation (CV), (ii) standard deviation (SD), and (iii) variability independent of the mean (VIM). VIM was calculated as 100 X SD/Mean^beta^, where beta is the regression coefficient based on the natural logarithm of the SD over the natural logarithm of the mean. The median number of cholesterol measurements per subject was 8.0 (interquartile range (IQR) 6.0–11.0).

### 2.3. Subgroups

Patients were stratified into quartiles (Q1, Q2, Q3, and Q4) based on the mean and variability (CV) of non-HDL-C levels. The ‘low mean’ and ‘high variability’ groups were operationally defined as the lowest (Q1) and highest (Q4) quartiles, respectively. The cohort was divided into four groups according to quartiles of the mean and variability of non-HDL-C: high mean/low variability (H/L, *n* = 263), high mean/high variability (H/H, *n* = 77), low mean/low variability (L/L, *n* = 76), and low mean/high variability (L/H, *n* = 37) (Figure 1).

### 2.4. Study Outcomes and Follow-Up

The endpoints of this study were newly diagnosed MI, stroke, or all-cause death. MI was defined as a hospitalization with ICD-10 codes of I21 or I22, or at least two claims with these codes. Stroke was defined as hospitalization with ICD-10 code I63 or I64, verified by brain magnetic resonance imaging or brain computed tomography. Patients were censored at the time of death or at the end of the study if they did not experience an endpoint event. The study population was followed from baseline to the date of death or cardiovascular event, or until 31 December 2020, whichever came first.

### 2.5. Statistical Analysis

All continuous variables are expressed as mean ± SD and were compared using an analysis of variance or a Kruskal–Wallis test, as appropriate. An independent *t*-test or Wilcoxon’s rank-sum test, followed by Bonferroni correction, was performed as a post hoc analysis. All categorical variables were compared using the chi-square test or Fisher’s exact test, as appropriate, and are expressed as numbers (proportion). The chi-square test or Fisher’s exact test, followed by Bonferroni correction, was performed as a post hoc analysis. The probability of MI, stroke, and all-cause mortality was evaluated using the Kaplan–Meier survival analysis and compared using the log-rank test. Cox proportional-hazards regression analyses were used to identify independent risk factors for the outcomes studied. Baseline clinical and laboratory parameters that demonstrated significant differences (*p* < 0.05) in univariable analysis or were known to affect outcomes were included in the multivariable model. All statistical analyses were performed using SAS software version 9.4. (SAS Institute Inc., Cary, NC, USA). A *p*-value < 0.005 was considered statistically significant.

## 3. Results

### 3.1. Baseline Characteristics of the Study Population

Table 1 summarizes the baseline clinical characteristics of the four mean and variability groups. Subjects in the higher quartiles of variability showed a higher prevalence of DM and hypertension and more frequent use of statins. The L/L group had the lowest BMI, and the L/H group had the lowest baseline serum albumin level. The H/H group had the highest baseline mean total cholesterol, triglyceride, and LDL-C levels. The mean non-HDL-C was low in the L/L and L/H groups, whereas the variability of non-HDL-C was high in the H/H and L/H groups, as defined. Age, dialysis vintage, follow-up period, and number of lipid profile measurements did not differ among the four groups.

### 3.2. Prediction of Myocardial Infarction

**Figure 2:**
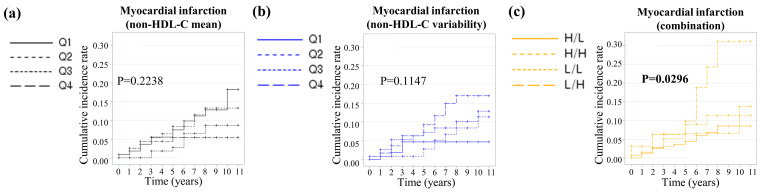
Kaplan–Meier estimates of myocardial infarction by (**a**) quartile of non-HDL cholesterol mean, (**b**) variability measured as the coefficient of variation, and (**c**) the combination of mean and variability.

During a median follow-up of 97.0 (IQR 73.0–127.0) months, 39 cases of MI occurred in the entire cohort. The incidence of MI was highest in the L/H group (*p* = 0.0346) (Table 2). When the cohort was divided into non-HDL-C mean or variability quartiles, the Kaplan–Meier curves showed that the cumulative MI incidence rate did not differ among groups (mean: log-rank *p* = 0.2238, variability: log-rank *p* = 0.1147) (Figure 2a,b). However, the cumulative MI incidence rate was significantly highest in the L/H group (log-rank *p* = 0.0296) (Figure 2c).

Univariable Cox regression analysis revealed that neither the mean nor variability (CV) of non-HDL-C per se was associated with MI (Table 3). However, age (hazard ratio (HR): 1.052, 95% confidence interval (CI): 1.023–1.081) and the L/H group were identified as independent risk factors for MI events (HR 3.355, 95% CI 1.401–8.035). After adjusting for age, sex, and mean non-HDL-C level, the associations between the risk of MI and age (HR: 1.053, 95% CI: 1.024–1.082) and between the risk of MI and the L/H group (HR: 3.311, 95% CI: 1.380–7.944) remained significant (Table 3).

### 3.3. Prediction of Stroke and All-Cause Mortality

**Figure 3:**
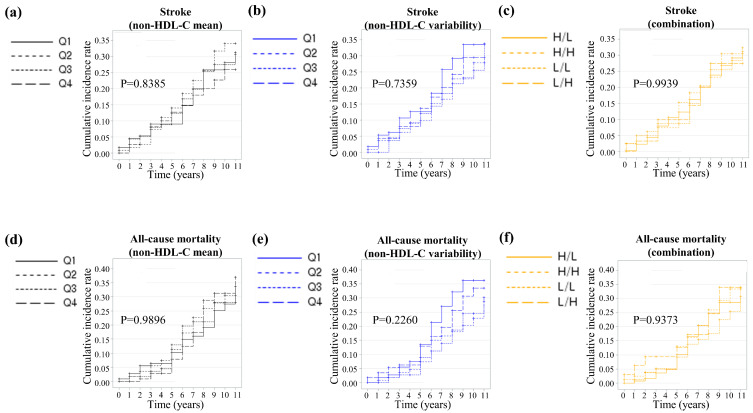
Kaplan–Meier estimates of (**a**–**c**) stroke and (**d**–**f**) all-cause mortality by quartile of non-HDL cholesterol mean, variability measured as coefficient of variation, and combination of mean and variability.

During the follow-up period, 99 cases of stroke were diagnosed and 96 deaths occurred. The incidence of stroke and all-cause mortality did not differ among the four groups, as shown in Table 2a–c. Additionally, the Kaplan–Meier curves also demonstrated that the cumulative incidence rates of stroke and all-cause mortality did not differ among the four groups, as depicted in Figure 3a–f.

Univariable Cox regression analysis showed that age was an independent risk factor for stroke (HR: 1.044, 95% CI: 1.027–1.062), and dialysis vintage was an independent protective factor against stoke (HR: 0.989, 95% CI: 0.980–0.997). Multivariable Cox regression, adjusted for age, sex, BMI, dialysis vintage, DM, hypertension, serum albumin, and the non-HDL combination group, revealed that age was an independent risk factor for stroke (HR: 1.040, 95% CI: 1.021–1.059) in our cohort (Table S1).

The univariable Cox regression analyses for all-cause mortality during follow-up showed that age (HR: 1.058, 95% CI: 1.040–1.077) and DM (HR: 1.674, 95% CI: 1.115–2.511) were independent risk factors, and dialysis vintage was an independent protective factor (HR: 0.870, 95% CI: 0.851–0.890). The multivariable Cox regression, adjusted for age, sex, BMI, dialysis vintage, DM, hypertension, serum albumin, and the non-HDL mean group, also demonstrated that age was an independent risk factor for all-cause death during follow-up (HR: 1.039, 95% CI: 1.019–1.059), and dialysis vintage remained an independent protective factor (HR: 0.873, 95% CI: 0.852–0.894) (Table S2).

## 4. Discussion

This study evaluated the association between non-HDL-C level and variability with the risks of CVD and all-cause death in patients undergoing HD. The findings suggest that, although the mean levels or variability of non-HDL-C alone are not significantly associated with MI, the combination of a low mean and high variability in non-HDL-C level is associated with an increased risk of MI. This finding may indicate that incorporating lipid fluctuations into risk assessment could provide additional insight beyond mean lipid levels alone.

Interestingly, at baseline, the proportion of patients taking statins was higher in the group with high variability. This might be because our analysis considered only statin use at baseline without assessing their ongoing adherence to treatment. Incomplete adherence to treatment could increase variability in cholesterol levels. Beyond noncompliance, factors such as polypharmacy and lack of clear guidelines for statin use in dialysis patients might also have influenced medication adherence and contributed to these findings.

An increase in non-HDL-C is associated with a higher risk of CVD and well reflects residual cardiovascular risk in the general population [12,13,14,15,16] and elevated levels of remnant cholesterol linked to plaque instability [17]. However, we found that a low mean of non-HDL-C combined with high variability was associated with increased MI risk in HD patients. In our cohort, HDL-C level did not differ significantly among the groups (Table 1), suggesting that low levels of non-HDL-C were primarily driven by low total cholesterol. In chronic conditions, such as ESKD requiring dialysis, low cholesterol levels are associated with poor outcomes. This phenomenon, known as the ‘*cholesterol paradox*’, arises from malnutrition, cachexia, and an increased burden of systemic inflammation [18,19,20,21]. Our findings support the validity of this explanation: patients in the L/H group had significantly lower albumin levels and total cholesterol, indicative of malnutrition. This nutritional imbalance could explain the increased MI risk observed in this group.

In addition to the low mean of non-HDL-C, high variability was an important factor for MI risk in our cohort. Variability in non-HDL-C, reflecting underlying metabolic instability or poor lipid control, was previously proposed as a potential marker of cardiovascular risk [22,23,24,25,26]. The independent association between L/H group and MI risk persisted even after adjusting for key confounders such as age, sex, and mean non-HDL-C levels. This suggests that high lipid variability might exacerbate vascular injury or promote atherogenesis by contributing to coronary plaque instability or rupture, potentially leading to MI [26,27,28,29]. Moreover, in patients with low mean non-HDL-C, poorer nutritional reserves may amplify the detrimental effect of lipid variability. Interestingly, the absence of a direct association between the mean or CV of non-HDL-C and MI underscores the importance of considering combined metrics of lipid variability and absolute levels when assessing cardiovascular risk.

On the other hand, unlike MI, the incidence of stroke and all-cause mortality did not differ significantly among the groups. Age was the most consistent independent predictor of both outcomes, highlighting its overriding role in long-term prognosis. Moreover, the lack of association between non-HDL-C profiles and stroke or all-cause mortality might reflect the multifactorial etiology of these outcomes in the dialysis population. Non-lipid factors, such as uremia-related inflammation, malnutrition, and vascular calcification, likely play a dominant role in driving these events, overshadowing the contribution of lipid variability.

This study has several limitations. First, with our data alone, it is not possible to determine whether reducing cholesterol variability lowers the risk of MI or if statin use reduces cholesterol variability. Therefore, the clinical implications of our findings are difficult to ascertain. Nevertheless, the significance of this study lies in the fact that, although the impact of absolute cholesterol values is inconsistent in dialysis patients, cholesterol variability is associated with cardiovascular event occurrence. Second, residual confounding effects from nutritional status, cardiovascular risk-modifying medications, and unmeasured variables, such as inflammation markers or detailed medication adherence, cannot be excluded. Moreover, because the number of incident MI events was only 39, we were unable to consider many factors such as comorbidities, statin usage, and dialysis vintage. To minimize bias, the quartile cutoffs were adjusted differently based on sex. A larger cohort is needed to confirm our findings.

Despite these limitations, it is crucial to recognize that HD patients present a unique cardiovascular risk profile. Dyslipidemia in ESKD is distinctively characterized by normal-to-low LDL-C, elevated triglycerides, and low HDL-C, differing from the general population [30,31]. While observational studies often report an inverse association between cholesterol and survival, this relationship is largely confounded by chronic inflammation and malnutrition [21,32]. Moreover, the ‘uremic milieu’ leads to the accumulation of highly atherogenic particles, which drive vascular calcification and plaque instability via mechanisms distinct from classic atherosclerosis [33,34]. Consequently, standard risk scores and static LDL targets may not fully capture this complex pathophysiology. In this context, our study offers significant clinical value. We demonstrated that a distinct phenotype—low mean non-HDL-C combined with high variability—is strongly associated with increased risk of myocardial infarction.

## 5. Conclusions

In conclusion, this study suggests that a low mean and high variability in non-HDL-C levels is associated with an increased risk of MI in HD patients, independent of other clinical characteristics. Stroke and all-cause mortality, on the other hand, are predominantly influenced by age, DM, and dialysis vintage. These findings highlight the potential value of a tailored, multifaceted approach to risk assessment and management in the vulnerable population of ESKD patients.

## Figures and Tables

**Table 1:** Clinical characteristics in the four groups based on the mean and variability (CV) of non-HDL cholesterol.

	High Mean /Low Variability (*n* = 258)	High Mean /High Variability (*n* = 82)	Low Mean /Low Variability (*n* = 81)	Low Mean /High Variability (*n* = 32)	*p*-Value
Age (years)	55.90 ± 12.99	57.55 ± 13.09	55.58 ± 13.72	56.59 ± 13.38	0.7983
Sex (male)	132 (51.16%)	40 (48.78%)	39 (48.15%)	18 (56.25%)	0.8627
BMI (kg/m^2^)	23.53 ± 4.01	24.74 ± 4.22	22.51 ± 4.22 *	23.46 ± 3.77	0.0038
Dialysis vintage (months)	23.22 ± 40.57	19.83 ± 46.39	30.14 ± 45.78	18.69 ± 27.73	0.1138
DM (%)	84 (32.56%)	41 (50.00%) *	27 (33.33%)	17 (53.13%)	0.0070
HTN (%)	58 (22.48%)	30 (36.59%)	21 (25.93%)	12 (37.50%)	0.0389
On statin (%)	65 (25.19%)	34 (41.46%) *	13 (16.05%) ^†^	12 (37.50%)	0.0013
Serum albumin (g/dL)	3.94 ± 0.40	3.81 ± 0.59	3.86 ± 0.46	3.62 ± 0.53 *	0.0121
CRP (mg/dL)	2.14 ± 8.03	8.59 ± 45.01	3.18 ± 10.33	0.76 ± 1.59	0.4155
Total cholesterol (mg/dL)	168.34 ± 39.93	204.19 ± 68.98 *	129.83 ± 26.87 *^†^	133.94 ± 34.35 *^†^	<0.001
Triglyceride (mg/dL)	140.03 ± 86.03	209.29 ± 158.64 *	105.68 ± 51.70 *^†^	102.55 ± 55.32 ^†^	<0.001
LDL-C (mg/dL)	95.95 ± 32.40	113.04 ± 50.78	63.25 ± 17.04 *^†^	64.43 ± 22.69 *^†^	<0.001
HDL-C (mg/dL)	38.96 ± 11.15	40.16 ± 14.63	38.90 ± 11.70	42.13 ± 11.17	0.4705
Non-HDL mean (mg/dL)	117.84 ± 24.69	119.78 ± 23.40	78.88 ± 13.08 *^†^	73.68 ± 12.39 *^†^	<0.001
Non-HDL-C variability					
CV (%)	19.50 ± 5.76	37.48 ± 11.05 *	19.12 ± 5.14 ^†^	38.08 ± 6.50 *^‡^	<0.001
VIM (%)	23.07 ± 8.94	44.74 ± 14.91 *	15.08 ± 4.75 *^†^	27.78 ± 5.07 *^†‡^	<0.001
ARV (mg/dL)	23.01 ± 10.65	36.78 ± 15.12 *	14.82 ± 6.36 *^†^	24.46 ± 8.13 ^†‡^	<0.001
Follow-up period (months)	95.50 [73.00–122.00]	94.00 [54.00–141.00]	107.00 [54.00–141.00]	106.00 [61.00–141.00]	0.2767
Number of lipid profile measurements	8.00 [6.00, 10.00]	9.00 [7.00, 11.00]	9.00 [6.00, 11.00]	9.00 [6.50, 11.00]	0.1071

Data are expressed as the mean ± standard deviation or *n* (%). BMI, body mass index; DM, diabetes mellitus; HTN, hypertension; LDL-C, low-density lipoprotein cholesterol; HDL-C, high-density lipoprotein cholesterol; CV, coefficient of variation; VIM, variability independent of the mean; ARV, average real variability.* *p* < 0.05 compared with H/L, ^†^ *p* < 0.05 compared with H/H, ^‡^ *p* < 0.05 compared with L/L.

**Table 2:** Incidence of MI, stroke, and all-cause death according to the mean and variability (CV) of non-HDL cholesterol.

(a) Mean	Q1 (*n* = 113)	Q2 (*n* = 112)	Q3 (*n* = 114)	Q4 (*n* = 114)	*p*-Value
MI	14 (12.39%)	12 (10.71%)	7 (6.14%)	6 (5.26%)	0.1585
Stroke	25 (22.12%)	28 (25.00%)	25 (21.93%)	21 (18.42%)	0.6960
All-cause death	24 (21.24%)	25 (22.32%)	24 (21.05%)	23 (20.18%)	0.9841
(b) Variability	Q1 (*n* = 113)	Q2 (*n* = 113)	Q3 (*n* = 113)	Q4 (*n* = 114)	*p*-value
MI	5 (4.42%)	8 (7.08%)	11 (9.73%)	15 (13.16%)	0.1101
Stroke	28 (24.78%)	23 (20.35%)	24 (21.24%)	24 (21.05%)	0.8536
All-cause death	30 (26.55%)	21 (18.58%)	20 (17.70%)	25 (21.93%)	0.3526
(c) Combination	High mean /Low variability (*n* = 258)	High mean /High variability (*n* = 82)	Low mean /Low variability (*n* = 81)	Low mean /High variability (*n* = 32)	*p*-value
MI	17 (6.59%)	8 (9.76%)	7 (8.64%)	7 (21.88%)	0.0346
Stroke	56 (21.71%)	18 (21.95%)	19 (23.46%)	6 (18.75%)	0.9589
All-cause death	54 (20.93%)	18 (21.95%)	17 (20.99%)	7 (21.88%)	0.9971

Data are expressed as *n* (%). CV, coefficient of variation; MI, myocardial infarction.

**Table 3:** Hazard ratio for MI according to the mean and variability (CV) of non-HDL cholesterol.

(a) Mean	Univariable			Multivariable		
	HR	95% C.I.	*p*-Value	HR	95% C.I.	*p*-Value
Age	1.053	1.024, 1.083	0.0003	1.054	1.024, 1.085	0.0003
Sex (male)	1.215	0.647, 2.281	0.5450	1.336	0.708, 2.521	0.3707
Q1	Reference			Reference		
Q2	0.881	0.436, 1.974	0.8453	0.879	0.413, 1.871	0.7372
Q3	0.509	0.207, 1.273	0.1504	0.522	0.210, 1.295	0.1609
Q4	0.445	0.171, 1.157	0.0965	0.458	0.176, 1.193	0.1098
(b) Variability	Univariable			Multivariable		
	HR	95% C.I.	*p*-value	HR	95% C.I.	*p*-value
Age	1.052	1.023, 1.081	0.0003	1.053	1.024, 1.082	0.0003
Sex (male)	1.275	0.684, 2.379	0.4443	1.478	0.785, 2.781	0.2261
Q1	Reference			Reference		
Q2	1.216	0.422, 3.505	0.7177	1.296	0.449, 3.743	0.6313
Q3	1.712	0.633, 4.630	0.2899	1.733	0.640, 4.695	0.2793
Q4	2.471	0.959, 6.369	0.0611	2.410	0.932, 6.231	0.0696
(c) Combination	Univariable			Multivariable		
	HR	95% C.I.	*p*-value	HR	95% C.I.	*p*-value
Age	1.052	1.023, 1.081	0.0003	1.053	1.024, 1.082	0.0003
Sex (male)	1.275	0.684, 2.379	0.4443	1.401	0.746, 2.631	0.2949
High mean/low variability	Reference			Reference		
High mean/high variability	1.439	0.626, 3.310	0.3919	1.335	0.577, 3.088	0.4991
Low mean/low variability	1.177	0.491, 2.821	0.7143	1.159	0.483, 2.780	0.7406
Low mean/high variability	3.355	1.401, 8.035	0.0066	3.311	1.380, 7.944	0.0073

CV, coefficient of variation; HR, hazard ratio; C.I., confidence interval; Q, quartile.

## Data Availability

The data presented in this study are available on request from the corresponding author.

## Supplementary Materials

The following supporting information can be downloaded at: https://www.mdpi.com/article/10.3390/jcm15010242/s1. Table S1: Hazard ratio for stroke according to the mean and variability (CV) of non-HDL cholesterol. Table S2: Hazard ratio for all-cause death during follow-up according to mean and variability (CV) of non-HDL cholesterol.

## Abbreviations

The following abbreviations are used in this manuscript:

BMI	Body mass index
CI	Confidence interval
CV	Coefficient of variation
CVD	Cardiovascular disease
DM	Diabetes mellitus
ESKD	End-stage kidney disease
HD	Hemodialysis
HR	Hazard ratio
ICD-10	International Classification of Disease, 10th Revision
IQR	Interquartile range
LDL-C	Low density lipoprotein cholesterol
MI	Myocardial infarction
Non-HDL-C	Non-high-density lipoprotein cholesterol
SD	Standard deviation
VIM	Variability independent of the mean
