# Supplementary material for: Combined Impact of Mean and Variability of Non-HDL Cholesterol on Myocardial Infarction in Hemodialysis Patients
Source: J Clin Med. 2025 Dec 28;15(1):242. doi: 10.3390/jcm15010242 (PMC12787241; doi:10.3390/jcm15010242)
Supplement: Supplementary file 1 [file jcm-15-00242-s001.zip › jcm-4002708-supplementary.pdf]

Supplemental Table S1. Hazard ratio for stroke according to the mean and variability (CV) of non-HDL cholesterol.

(A) Mean	Univariable			Multivariable		
	HR	95% C.I.	p-value	HR	95% C.I.	p-value
**Age**	**1.044**	**1.027, 1.062**	**<0.0001***	**1.040**	**1.021, 1.059**	**<0.0001***
Sex (male)	1.001	0.675, 1.485	0.9968	1.020	0.674, 1.543	0.9272
BMI	0.988	0.942, 1.036	0.6120	0.984	0.932, 1.038	0.5425
**Dialysis vintage**	**0.989**	**0.980, 0.997**	**0.0090***	0.993	0.984, 1.002	0.1357
DM	1.390	0.926, 2.086	0.1120	1.192	0.734, 1.937	0.4779
HTN	1.474	0.963, 2.257	0.0743	1.334	0.802, 2.218	0.2667
On statin	1.116	0.706, 1.762	0.6386	0.768	0.444, 1.329	0.3461
Serum albumin	0.790	0.520, 1.199	0.2678	0.848	0.538, 1.337	0.4781
Q1	Reference					
Q2	1.129	0.658, 1.936	0.6603	1.050	0.600, 1.837	0.8638
Q3	1.045	0.600, 1.821	0.8751	1.111	0.624, 1.977	0.7204
Q4	0.897	0.502, 1.604	0.7144	0.814	0.435, 1.524	0.5205

(B) Variability	Univariable			Multivariable		
	HR	95% C.I.	p-value	HR	95% C.I.	p-value
**Age**	**1.044**	**1.027, 1.062**	**<0.0001***	**1.040**	**1.021, 1.059**	**<0.0001***
Sex (male)	1.001	0.675, 1.485	0.9968	1.024	0.677, 1.548	0.9113
BMI	0.988	0.942, 1.036	0.6120	0.981	0.931, 1.034	0.4782
**Dialysis vintage**	**0.989**	**0.980, 0.997**	**0.0090***	0.994	0.985, 1.003	0.2102
DM	1.390	0.926, 2.086	0.1120	1.248	0.763, 2.043	0.3778
HTN	1.474	0.963, 2.257	0.0743	1.298	0.783, 2.150	0.3117
On statin	1.116	0.706, 1.762	0.6386	0.790	0.458, 1.364	0.3973
Serum albumin	0.790	0.520, 1.199	0.2678	0.846	0.540, 1.327	0.4667
Q1	Reference			Reference		
Q2	0.742	0.427, 1.288	0.2884	0.772	0.435, 1.372	0.3782
Q3	0.790	0.458, 1.363	0.3965	0.779	0.438, 1.388	0.3976
Q4	0.850	0.493, 1.466	0.5585	0.810	0.457, 1.434	0.4692

(C) Combination	Univariable			Multivariable		
	HR	95% C.I.	p-value	HR	95% C.I.	p-value
**Age**	**1.044**	**1.027, 1.062**	**<0.0001***	**1.040**	**1.021, 1.059**	**<0.0001***
Sex (male)	1.001	0.675, 1.485	0.9968	1.031	0.681, 1.561	0.8841
BMI	0.988	0.942, 1.036	0.6120	0.982	0.931, 1.035	0.4903
**Dialysis vintage**	**0.989**	**0.980, 0.997**	**0.0090***	0.993	0.984, 1.003	0.1677
DM	1.390	0.926, 2.086	0.1120	1.230	0.758, 1.998	0.4020
HTN	1.474	0.963, 2.257	0.0743	1.322	0.798, 2.193	0.2786
On statin	1.116	0.706, 1.762	0.6386	0.775	0.450, 1.335	0.3580
Serum albumin	0.790	0.520, 1.199	0.2678	0.831	0.528, 1.308	0.4242
High mean/Low variability	Reference			Reference		

High mean/High variability	1.043	0.613, 1.774	0.8776	0.997	0.570, 1.743	0.9905
Low mean/Low variability	1.001	0.595, 1.686	0.9956	1.043	0.608, 1.790	0.8786
Low mean/High variability	0.934	0.402, 2.167	0.8730	0.884	0.371, 2.108	0.7808

Supplemental Table S2. Hazard ratio for all-cause death during follow up according to the mean and variability (CV) of non-HDL cholesterol

(A) Mean	HR	Univariable<br>95% C.I.	p-value	HR	Multivariable<br>95% C.I.	p-value
**Age**	**1.058**	**1.040, 1.077**	**<0.0001***	**1.039**	**1.019, 1.059**	**<0.0001***
Sex (male)	1.014	0.681, 1.510	0.9449	0.873	0.569, 1.339	0.5342
BMI	1.016	0.969, 1.065	0.5197	1.013	0.956, 1.073	0.6627
**Dialysis vintage**	**0.870**	**0.851, 0.890**	**<0.0001***	**0.873**	**0.852, 0.894**	**<0.0001***
**DM**	**1.674**	**1.115, 2.511**	**0.0129***	0.910	0.561, 1.477	0.7030
HTN	1.533	0.995, 2.362	0.0529	1.397	0.824, 2.368	0.2148
On statin	1.116	0.690, 1.807	0.6543	0.641	0.357, 1.151	0.1363
Serum albumin	0.674	0.448, 1.015	0.0590	0.776	0.479, 1.256	0.3021
Q1	Reference			Reference		
Q2	1.217	0.703, 2.105	0.4831	1.557	0.857, 2.829	0.1458
Q3	1.049	0.591, 1.864	0.8696	1.355	0.719, 2.554	0.3477
Q4	1.216	0.689, 2.145	0.4995	0.883	0.471, 1.652	0.6963

(B) Variability	HR	Univariable<br>95% C.I.	p-value	HR	Multivariable<br>95% C.I.	p-value
**Age**	**1.058**	**1.040, 1.077**	**<0.0001***	**1.039**	**1.020, 1.059**	**<0.0001***
Sex (male)	1.014	0.681, 1.510	0.9449	0.872	0.567, 1.340	0.5318
BMI	1.016	0.969, 1.065	0.5197	1.016	0.960, 1.076	0.5845
**Dialysis vintage**	**0.870**	**0.851, 0.890**	**<0.0001***	**0.876**	**0.855, 0.897**	**<0.0001***
**DM**	**1.674**	**1.115, 2.511**	**0.0129***	0.976	0.603, 1.581	0.9217
HTN	1.533	0.995, 2.362	0.0529	1.312	0.771, 2.231	0.3165
On statin	1.116	0.690, 1.807	0.6543	0.692	0.387, 1.236	0.2133
Serum albumin	0.674	0.448, 1.015	0.0590	0.749	0.470, 1.196	0.2262
Q1	Reference			Reference		
Q2	0.639	0.370, 1.103	0.1079	0.955	0.530, 1.720	0.8785
Q3	0.532	0.300, 0.942	0.0303*	0.792	0.438, 1.432	0.4400
Q4	0.751	0.443, 1.272	0.2867	0.786	0.444, 1.394	0.4107

(C) Combination	HR	Univariable<br>95% C.I.	p-value	HR	Multivariable<br>95% C.I.	p-value
**Age**	**1.058**	**1.040, 1.077**	**<0.0001***	**1.041**	**1.022, 1.061**	**<0.0001***
Sex (male)	1.014	0.681, 1.510	0.9449	0.939	0.610, 1.444	0.7730
BMI	1.016	0.969, 1.065	0.5197	1.010	0.954, 1.070	0.7209
**Dialysis vintage**	**0.870**	**0.851, 0.890**	**<0.0001***	**0.870**	**0.849, 0.893**	**<0.0001***
**DM**	**1.674**	**1.115, 2.511**	**0.0129***	0.934	0.578, 1.507	0.7787
HTN	1.533	0.995, 2.362	0.0529	1.320	0.782, 2.228	0.2981
On statin	1.116	0.690, 1.807	0.6543	0.654	0.366, 1.169	0.1520
Serum albumin	0.674	0.448, 1.015	0.0590	0.689	0.428, 1.111	0.1268
High mean/Low variability	Reference			Reference		

High mean/High variability	1.043	0.613, 1.773	0.8776	1.138	0.645, 2.006	0.6559
Low mean/Low variability	0.843	0.489, 1.453	0.5390	1.090	0.613, 1.938	0.7689
Low mean/High variability	0.943	0.430, 2.069	0.8837	0.452	0.173, 1.182	0.1054
